# Efficient compartmentalization in insect bacteriomes protects symbiotic bacteria from host immune system

**DOI:** 10.1186/s40168-022-01334-8

**Published:** 2022-09-27

**Authors:** Mariana Galvão Ferrarini, Elisa Dell’Aglio, Agnès Vallier, Séverine Balmand, Carole Vincent-Monégat, Sandrine Hughes, Benjamin Gillet, Nicolas Parisot, Anna Zaidman-Rémy, Cristina Vieira, Abdelaziz Heddi, Rita Rebollo

**Affiliations:** 1 Univ Lyon, INRAE, INSA-Lyon, BF2I, UMR 203, 69621 Villeurbanne, France; 2 Laboratoire de Biométrie et Biologie Evolutive, UMR5558, Université Lyon 1, Université Lyon, Villeurbanne, France; 3 Univ Lyon, INSA-Lyon, INRAE, BF2I, UMR 203, 69621 Villeurbanne, France; 4 UMR5242, Institut de Génomique Fonctionnelle de Lyon (IGFL), Ecole Normale Supérieure de Lyon, Centre National de la Recherche Scientifique (CNRS), Université Claude Bernard Lyon 1 (UCBL), Université de Lyon (Univ Lyon), F-69007 Lyon, France

**Keywords:** Symbiosis, Immunity, Bacteria, Antimicrobial peptides, Coleoptera, TCT

## Abstract

**Background:**

Many insects house symbiotic intracellular bacteria (endosymbionts) that provide them with essential nutrients, thus promoting the usage of nutrient-poor habitats. Endosymbiont seclusion within host specialized cells, called bacteriocytes, often organized in a dedicated organ, the bacteriome, is crucial in protecting them from host immune defenses while avoiding chronic host immune activation. Previous evidence obtained in the cereal weevil *Sitophilus oryzae* has shown that bacteriome immunity is activated against invading pathogens, suggesting endosymbionts might be targeted and impacted by immune effectors during an immune challenge. To pinpoint any molecular determinants associated with such challenges, we conducted a dual transcriptomic analysis of *S. oryzae*’s bacteriome subjected to immunogenic peptidoglycan fragments.

**Results:**

We show that upon immune challenge, the bacteriome actively participates in the innate immune response via induction of antimicrobial peptides (AMPs). Surprisingly, endosymbionts do not undergo any transcriptomic changes, indicating that this potential threat goes unnoticed. Immunohistochemistry showed that TCT-induced AMPs are located outside the bacteriome, excluding direct contact with the endosymbionts.

**Conclusions:**

This work demonstrates that endosymbiont protection during an immune challenge is mainly achieved by efficient confinement within bacteriomes, which provides physical separation between host systemic response and endosymbionts.

Video Abstract

**Supplementary Information:**

The online version contains supplementary material available at 10.1186/s40168-022-01334-8.

## Background

Nutritional symbiosis between animals and microorganisms is a major driver of adaptation [1] as it participates in the colonization of nutrient-poor environments by complementing the metabolic needs of the host [2]. Notably, thanks to intracellular symbiotic bacteria (endosymbionts), insects can thrive on unbalanced carbohydrate-based diets, including blood, plant sap, or cereal grains [1, 3–6]. However, the constant presence of microorganisms within an insect’s body represents a permanent challenge for the immune system [7]. The host immune system must conserve its ability to react against pathogens, while keeping beneficial symbionts alive and metabolically active [8]. The establishment of an equilibrium between excessive host colonization by the symbiont and chronic activation of the host immune system is essential in such symbiotic relationships, as the former would be detrimental to host survival, while the latter would result in symbiotic damage and host fitness reduction [9]. To better understand the co-evolution between the host immune system and the intracellular symbiotic bacteria, it is therefore important to pinpoint the molecular determinants of endosymbiont tolerance and pathogen control.

The association between the cereal weevil *Sitophilus oryzae* and its recently acquired Gram-negative intracellular bacterium, *Sodalis pierantonius* (~ 28K years [10, 11]), is a remarkable example of homeostasis between insects and endosymbionts. *S. pierantonius* are contained within specialized gigantic cells, the bacteriocytes, which at the larval stages are located in a specialized organ—the bacteriome—at the foregut-midgut junction [3, 12]. While wild *S. oryzae* animals are always associated with *S. pierantonius*, comparative studies between symbiotic and artificially obtained aposymbiotic insects have shown that the presence of the endosymbiont accelerates insect development, allows strengthening of the insect cuticle [13], and enables flying [14].

Contrary to most long-lasting insect endosymbionts, the *S. pierantonius* genome contains genes encoding a functional type III secretion system (T3SS) [15], which was shown to be necessary during insect metamorphosis, where host stem cells are infected by the endosymbiont, followed by bacteriocyte differentiation and adult bacteriome formation [16]. The *S. pierantonius* genome also encodes genes necessary for microbial-associated molecular pattern (MAMP) synthesis, including peptidoglycans (PGs), which are able to activate the insect immune responses through their interaction with host pattern recognition receptors [7]. Injection of *S. pierantonius* into the insect hemolymph triggers the production of a plethora of antimicrobial peptides (AMPs) [17], suggesting its presence within the host body is an ongoing immune threat. Nevertheless, chronic immune system activation is avoided by the compartmentalization of the endosymbiont within bacteriocytes and the expression of an adapted local immune system [17–20]. The coleoptericin A (ColA) antimicrobial peptide (AMP) is an important molecular determinant for the maintenance of *S. oryzae/S. pierantonius* homeostasis. By interacting with the bacterial chaperonin GroEL, ColA inhibits bacterial cell septation and generates elongated bacteria with multiple genome copies [18]. Inhibition of *colA* with RNA interference leads to bacterial escape from the bacteriome, and colonization of host surrounding tissues [18]. ColA expression in the bacteriome is dependent on *relish* and *imd*, two genes belonging to the immune deficiency (IMD) pathway [21]. Recently, the weevil’s peptidoglycan recognition protein LB (PGRP-LB) was also shown to play a central role in host homeostasis. By cleaving the tracheal cytotoxin (TCT), a monomeric form of DAP-type peptidoglycan constantly produced by the endosymbionts within the bacteriome, PGRP-LB prevents the exit of TCT from the bacteriome to the insect’s hemolymph, avoiding a chronic activation of host IMD-dependent humoral immunity [19]. Taken together, these results suggest that bacterial compartmentalization in the bacteriome is a key strategy that allows the tolerance of symbiotic bacteria as it avoids the contact between the endosymbionts and the insect’s immune system [22], therefore preventing chronic activation of the host immune IMD pathway against the beneficial microorganisms [23].

Current knowledge of gene expression levels in the larval bacteriome is limited to a couple of AMPs and a few other stress-related insect genes [19–21], and little is known about other insect or bacterial regulatory mechanisms involved in endosymbiont protection from bacteriocyte immune activation. We have previously shown that the bacteriome participates in the immune response against pathogenic bacteria and TCT challenge. Notably, upregulation of several AMPs in weevils after injection of bacteria into the insect hemolymph is observed in the bacteriome [17, 20], as well as in the rest of the body [17, 20, 24]. In addition, TCT injection is sufficient to mimic AMP induction in larval bacteriomes upon bacterial challenge [19]. It is important to note that AMP induction upon TCT challenge is IMD-dependent, as is the control of endosymbionts within bacteriocytes, indicating the same pathway can fight exogenous bacterial infection while controlling intracellular beneficial bacteria [21]. Although the involvement of the bacteriome in the immune response would appear in disagreement with its primary function of hosting bacteria, such activation of the immune response against external infections does not seem to pose a threat to *S. pierantonius* integrity since bacterial infections do not induce a reduction in the number of symbionts [20]. This suggests that, despite activating the same immune pathway, differences must exist between fighting external infections and protecting the intracellular symbiont. We hypothesize that either the endosymbionts have evolved specific mechanisms to counteract the bacteriome immune response or that host-controlled mechanisms, such as AMP secretion, ensure endosymbiont protection.

In this work, we conducted a global dual transcriptomic analysis of host  bacteriomes and bacteria challenged systemically with TCT, in order to mimic an immune response in the absence of a real infectious threat. While confirming the involvement of the bacteriome in the immune response, notably via an AMP induction, immunohistochemical observations showed AMP accumulation only outside of the bacteriome and a full preservation of the basal bacterial transcriptional program. Thus, efficient physical separation between symbionts and bacteria-harnessing molecules ensures full symbiont protection during an immune challenge.

## Methods

### Animal rearing, peptidoglycan challenge, and sample preparation

*S. oryzae* laboratory strain (Bouriz) was reared on wheat grains at 27.5 °C and at 70% relative humidity. A strain of aposymbiotic insects was obtained as previously described [25]. The DAP-type peptidoglycan fragment TCT was purified from *Escherichia coli* as previously described [26]. Fourth instar larvae were extracted from wheat grains and challenged with a 0.2-mM TCT solution diluted in 1× phosphate-buffered saline (PBS) injected into the hemolymph using a Nanoject III (Drummond). Sterile phosphate-buffered saline PBS was also used as a negative control. Injected and non-injected larvae (naïve) were kept in white flour for 6 h at 27.5 °C and at 70% relative humidity before dissection. Bacteriomes were dissected in diethylpyrocarbonate-treated buffer A (25 mM KCl, 10 mM MgCl_2_, 250 mM sucrose, 35 mM Tris/HCl, pH = 7.5). For each sample, bacteriomes were pooled (30 for dual RNA-seq library preparation, and at least 25 for RT-qPCR) and stored at − 80 °C prior to RNA extraction. Pools of five carcasses from symbiotic dissected weevils were used for RT-qPCR. Aposymbiotic samples consisted of pools of five fourth-instar aposymbiotic larvae, which were torn in buffer A, but not dissected as they do not harbor bacteriomes.

### RNA extraction, library preparation, and sequencing

Total RNA was extracted with TRIzol™ Reagent (Invitrogen, ref.: 15596026) following the manufacturer’s instructions. Nucleic acids were then purified using the NucleoSpin RNA Clean up kit (Macherey Nagel, ref.: 740948). Genomic DNA was removed from the samples with the DNA-free DNA removal kit (Ambion, ref.: AM1906). Total RNA concentration and quality were checked using the Qubit Fluorometer (Thermo Fisher Scientific) and Tapestation 2200 (Agilent Biotechnologies). Ribo-depletion and dual RNA-seq strand-specific cDNA libraries were obtained starting from 100 ng of total RNA using the Ovation Universal RNA-seq System (NuGEN) following the manufacturer’s instructions. Libraries were sequenced on a Nextseq 500 sequencer (Illumina), using the NextSeq 500/550 High Output Kit (Illumina).

### Preprocessing, mapping of reads, and differential expression analysis

Raw reads were processed using Cutadapt v1.18 [27] to remove adapters and filter out reads shorter than 50 bp and reads that had a mean quality value lower or equal to 30. Clean reads were mapped against the *S. oryzae* genome (Genbank: PRJNA431034) with STAR v2.7.3a [28] and against the S*. pierantonius* genome (Genbank: CP006568.1) with Bowtie 2 v2.3.5 [29] with default parameters. Shared reads between the two genomes were filtered out with the aid of SAMtools v1.10 [30] and Picard v2.21.6 (available from https://broadinstitute.github.io/picard/). Gene counts were obtained for uniquely mapped reads with featureCounts v1.6.4 method from the Subread package [31]. Whenever uniquely mapped read counts were set to zero due to duplicated regions or multi-mapped reads, we further verified these regions within the multi-mapped read counts available with featureCounts. Insertion sequence (IS) families from the bacteria were also counted with the use of TEtools (v1.0.0) with default parameters [32]. Gene counts and TEtools counts were used as input for differential expression analyses using the DESeq2 v1.26.0 [33] package in R. After testing, the *p*-values were adjusted with the Benjamini-Hochberg correction [34] for multi-testing. Genes were considered differentially expressed when adjusted *p*-values (p-adj) were smaller than 0.05. Sequencing data from this study have been deposited at the National Center for Biotechnology Information Sequence Read Archive, https://www.ncbi.nlm.nih.gov/sra (accession no. PRJNA816415).

### Quantitative RT-PCR

Total RNA was extracted from fourth-instar bacteriomes and carcasses, as well as from whole aposymbiotic fourth-instar larvae using the RNAqueous - Micro kit (Ambion). DNA was removed with DNAse treatment, and RNA quality was checked with Nanodrop (Thermo Fisher Scientific). Complementary DNA (cDNA) was produced with the iScript™ cDNA Synthesis Kit (Bio-Rad) following the manufacturer’s instructions and starting with 500 ng total RNA. Differential gene expression was assessed by quantitative real-time PCR with a CFX Connect Real-Time PCR Detection System (Bio-Rad) using the LightCycler Fast Start DNA Master SYBR Green I kit (Roche Diagnostics), as previously described [19], except for *dpt4*, for which the annealing temperature was reduced to 54.5 °C. Data were normalized using the ratio of the target cDNA concentration to the geometric average of two housekeeping transcripts: *glyceraldehyde 3-phosphate dehydrogenase* (LOC115881082) and *malate oxidase* (LOC115886866). Primers were designed to amplify fragments of approximately 150 bp. A complete list of primers can be found in Additional file 3: Table S1.

### Immunohistochemistry

Larval samples challenged with TCT or PBS were prepared for histological observations as described in [19]. Briefly, samples were fixed in paraformaldehyde (PFA) 4%. After 1 day, the fixative was replaced by several washing with PBS before embedding the tissue in 1.3% agar then dehydrated through a gradient of ethanol (EtOH) washes and transferred to butanol-1, at 4°C, overnight. Samples were then placed in melted Paraplast, and 3-μm-thick sections were cut with a HM 340 E microtome (Thermo Fisher Scientific). Sections were placed on poly-lysine-coated slides, dried overnight at 37 °C, and stored at 4 °C.

For AMP localization, samples were dewaxed twice in methylcyclohexane for 10 min, rinsed in EtOH 100°, rehydrated through an EtOH gradient, and then placed in PBS with 1% bovine serum albumin (BSA) for 30 min. ColA rabbit primary polyclonal anti-serum [18] at 1:200 dilution and a Coleoptericin B (ColB) primary polyclonal anti-serum (Proteogenix, Schiltigheim-France) at 1:300 dilution in 0.1% BSA were used. Preimmune rabbit serum (J0) was used as a negative control for ColA anti-serum and BSA 0.1% for ColB (purified antibody). Antibody specificity was checked by western blot. After 1 h incubation at room temperature in the dark, the sections were washed with PBS containing 0.2% Tween. Samples were then incubated with anti-rabbit IgG, labeled with Alexa Fluor 488. This secondary antibody was applied for 1 h at room temperature, diluted at 1:500 in 0.1% BSA in PBS. The excess of secondary antibody was washed with PBS-Tween, rinsed with PBS, and washed several times with tap water. The sections were then dried and mounted using PermaFluor™ Aqueous Mounting Medium (Thermo Fisher Scientific), together with 4,6-diamidino-2-phenylindole (DAPI, Sigma-Aldrich) for nuclear staining (3 μg/ml of medium). Images were acquired using an epifluorescence microscope (Olympus IX81), under specific emission filters: HQ535/50 for the green signal (antibody staining), D470/40 for the blue signal (DAPI), and HQ610/75 for the red signal (unspecific autofluorescence from tissue). Images were captured using an XM10 camera and the CellSens Software (Soft Imaging System). Images were treated using ImageJ (release 1.47v).

## Results and discussion

### Dual RNA sequencing successfully yielded both insect and bacterial transcripts

**Fig. 1:**
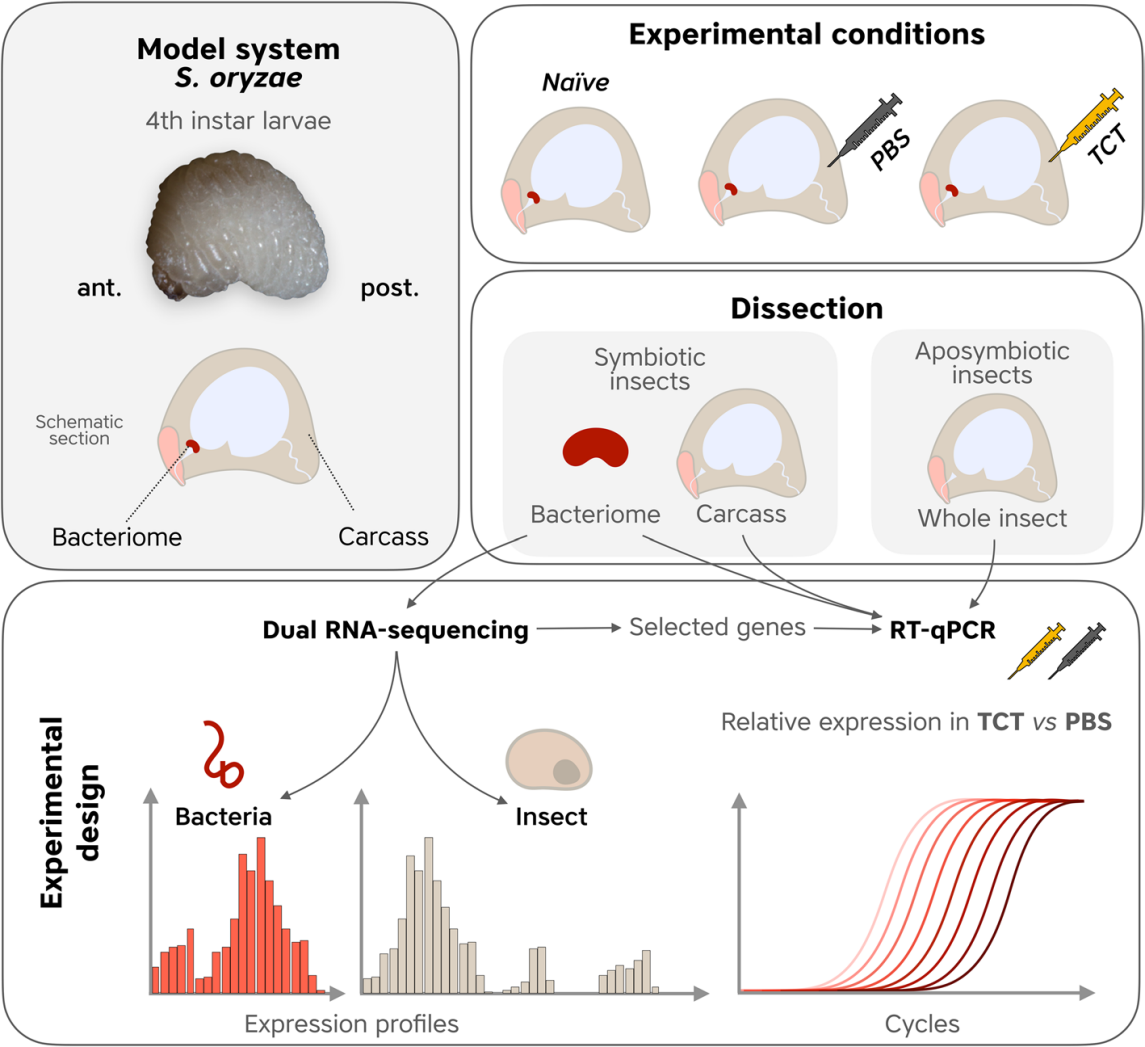
Schematic diagram of the experimental design. Top left panel: image of a *S. oryzae* 4th instar larva along with a schematic section. Top left panels: symbiotic and aposymbiotic *S. oryzae* fourth instar larvae were extracted from grains and dorsally injected with 0.2 mM PBS and 0.2 mM TCT at the level of the hemolymph. Other larvae were extracted from grains but not injected (naïve). Bacteriomes and carcasses were sampled from PBS/TCT-injected or naïve symbiotic larvae alongside whole aposymbiotic larvae. Bottom panel: dual RNA-seq was performed to detect insect, and bacterial expression profiles were performed on bacteriomes and carcasses of symbiotic weevils (PBS, TCT, and naïve samples). RT-qPCR experiments were performed on TCT- and PBS-treated bacteriomes/carcasses from symbiotic weevils as well as whole larvae from aposymbiotic weevils, to detect bacteriome-specific and/or symbiont-dependent transcriptomic changes

To investigate the bacteriome response to an immune challenge, we extracted *S. oryzae*’s fourth-instar larvae (L4) from grains and injected them with TCT, a fragment of the DAP-type peptidoglycan produced by Gram-negative bacteria, including *S. pierantonius* [19] and recovered bacteriomes 6 h post-injection as previously described [20]. TCT injection is able to trigger a potent response without the interference of an exogenous infectious bacteria [21]. Control larvae were injected with PBS or extracted from grains but not injected (see Fig. 1). To obtain the transcriptomic profile of both the symbiont and the host, dual RNA-seq was performed in triplicates and yielded from 105 to 140 M reads per library (Additional file 3: Table S2). The reads were cleaned from adapter sequences and low-quality reads, and around 85% of the raw reads were kept for further analyses. We subsequently mapped the clean reads against both genomes and obtained ~ 65–80% unambiguously mapping to the genome of *S. oryzae*, and ~ 5–8% to the genome of *S. pierantonius*. In each library, from 23 to 33 M, reads were uniquely mapped against insect genes (Additional file 3: Table S3), whereas ~ 3 M reads were uniquely mapped against bacterial genes (Additional file 3: Table S4). These results depict an improvement from our previous study, which yielded ~ 0.4 M reads mapped against bacterial genes in the same developmental stage and similar sequencing depth [16].

### Systemic TCT challenge primarily triggers AMP induction within the bacteriome

Sixteen *S. oryzae* genes were detected as differentially expressed (DE; *p*-adj < 0.05) 6 h after the TCT challenge in the bacteriome, with respect to the bacteriome of non-injected (naïve) or PBS-injected larvae (Table 1, Additional file 3: Table S5 and S4). Among these, one gene was strongly downregulated, four were mildly downregulated, and eleven were upregulated in response to TCT.

**Table 1:** *S. oryzae* genes differentially expressed in TCT-challenged bacteriomes (*p*-adj < 0.05) identified by dual RNA-seq

Gene information				Average expression (TPM)^b^			Log2 fold change	
Gene ID	Type	Gene abbreviation	Protein name	Naïve	PBS	TCT	TCT vs naïve	TCT vs PBS
*LOC115882681*	Unknown	N/A	Uncharacterized	2.93	1.06	0.03	− 6.941	− 5.574
*LOC115888453*	Transcription factor	*adf-1*	Transcription factor Adf-1 family	54.55	49.23	34.33	− 0.696	− 0.601
*LOC115881033*	Translation initiation	*eif4ebp-2*	Eukaryotic translation initiation factor 4E-binding protein 2	256.09	249.36	162.63	− 0.691	− 0.706
*LOC115891903*	Transcription factor	*nrbp*	Nuclear receptor-binding protein	117.12	128.47	83.03	− 0.518	− 0.600
*LOC115883362*	Transcription factor	*znf-91*	Zinc finger protein 91-like	48.09	46.24	36.16	− 0.436	− 0.434
*LOC115877563*	ABC transporter	*mrp-4*	Multidrug resistance-associated protein 4-like	1.41	1.30	3.44	1.277	1.335
*LOC115885681*	Growth factor	*brx*	Barietin toxin	1.24	1.13	4.30	1.793	1.881
*LOC115886735*	Bacterial recognition	*gnbp-1*	Beta-1,3-glucan-binding protein-like	7.07	8.72	37.97	2.411	2.052
*LOC115874620*	AMP	*col-A*	Coleoptericin-A	251.44	480.30	2116.35	3.030	2.040
*LOC115883884*	AMP	*lux*	Luxuriosin	7.34	6.01	60.94	3.042	3.256
*LOC115884866*	AMP	*glyr-amp*	Glycine-rich AMP	11.10	12.88	120.87	3.410	3.165
*LOC115888387*	AMP	*srx*	Sarcotoxin	27.81	28.61	407.20	3.826	3.734^a^
*LOC115877462*	AMP	*dpt-2*	Diptericin-2	40.45	63.50	731.07	4.131	3.425
*LOC115877463*	AMP	*dpt-3*	Diptericin-3	9.45	13.71	261.58	4.759	4.164
*LOC115874703*	AMP	*col-B*	Coleoptericin-B	1.97	3.42	86.46	5.386	4.538
*LOC115877465*	AMP	*dpt-4*	Diptericin-4	1.31	2.52	98.43	6.196	5.225

^a^This transcript was below the significance of detection in one of the conditions due to an outlier; results were verified with EdgeR and we validated this as a DE gene after the qPCRs

^b^Average expression is provided in transcripts per million (TPM)

**Fig. 2:**
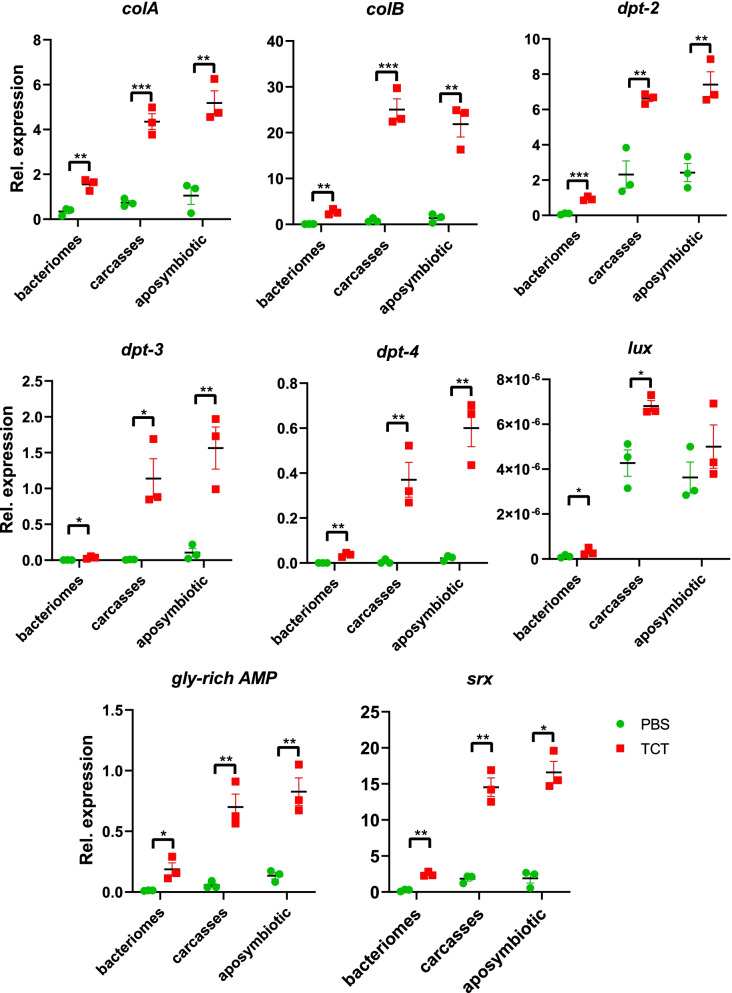
Differential expression of TCT-induced AMPs in bacteriomes. The quantification was performed by RT-qPCR on *S. oryzae* bacteriomes and carcasses of symbiotic weevils, as well as on whole aposymbiotic larvae. Green dots: PBS-injected larvae (control); red squares: TCT-injected larvae. Asterisks denote statistical significance (ANOVA with Kruskal-Wallis test, *p ≤ 0.05). Error bars represent SE. Overall, the AMP induction in response to TCT is observed in both bacteriomes and carcasses of symbiotic weevils, as well as in aposymbiotic weevils

**Fig. 3:**
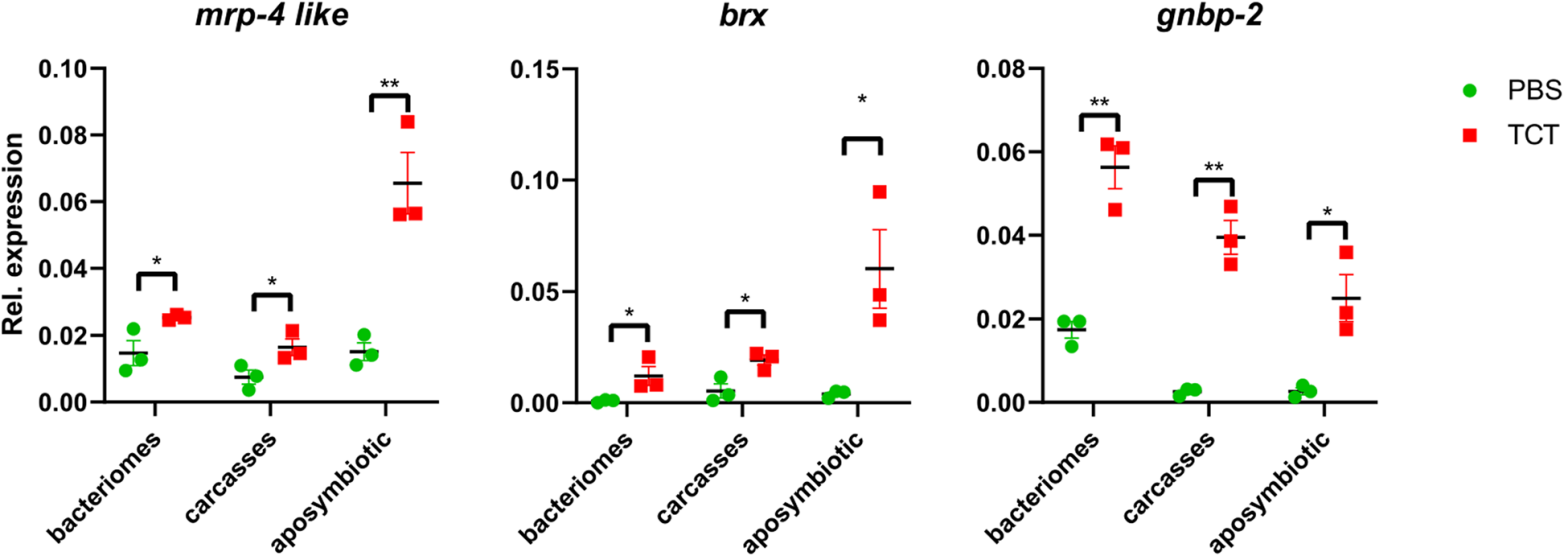
Differential expression of TCT-induced genes in bacteriomes other than AMPs. The quantification was performed by RT-qPCR on *S. oryzae* bacteriomes and carcasses of symbiotic weevils, as well as on whole aposymbiotic larvae. Green dots: PBS-injected larvae (control); red squares: TCT-injected larvae. Asterisks denote statistical significance (ANOVA with Kruskal-Wallis test, *p ≤ 0.05). Error bars represent SE. Overall, upregulation in response to TCT is observed in both bacteriomes and carcasses of symbiotic weevils, as well as in aposymbiotic weevils

RT-qPCR experiments confirmed the TCT-dependent induction of all 11 upregulated genes (Fig. 2, Additional file 1: Fig. S1). Eight of these genes encode AMPs, and all possess a predicted signal peptide: *colA* (Coleoptericin A), Coleoptericin B (*colB*), Sarcotoxin (*srx*), Luxoriosin (*lux*), a Gly-rich AMP (*gly-rich AMP*), and three Diptericins (*dpt-2*, *dpt-3*, and *dpt-4*, Fig. 2) [35]. This AMP induction is in agreement with previous reports, where AMPs induced in larvae by immune challenge included *colA* [17, 20, 21, 24], *colB*, *srx* [20, 21, 24], *dpt*, *cecropin*, and *defensins* [20, 24]. In addition to the eight AMPs, genes encoding one Gram-negative binding protein (*gnbp-2*), a barietin-like toxin (*brx*), and a multidrug-resistant protein (*mrp-4*) were also upregulated in the bacteriome (Fig. 3). These three genes have not been identified in previous studies. *gnbp-2* is likely involved in insect defense responses against Gram-negative bacteria [36, 37] and, like AMPs, contains a predicted secretory sequence at the peptide N-terminus (SignalP 6.0 likelihood value of 0.9998). It is noteworthy that another member of the *gnbp-2* family was also shown to be upregulated in *S. oryzae* bacteriome in response to a bacterial challenge in a previous study [24]. The barietin-like toxin likely acts as a toxin directed against bacteria [38], similarly to AMPs, and also contains a predicted secretory sequence in the N-terminal region (SignalP 6.0 likelihood value of 1.0). Finally, *mrp-4 like* is likely a transporter involved in the secretion of toxins and/or regulating homeostasis against pathogens [39]. In contrast, none of the downregulated bacteriome genes detected in the Dual RNA-seq was confirmed by RT-qPCR (Additional file 1: Fig. S1). These results might be explained by their less pronounced downregulation as seen by a milder Log2FC. Moreover, dual RNA-seq was obtained from total ribodepleted RNAs, while RT-qPCR was performed on polyadenylated mRNAs, which could contribute to the differences observed in these analyses.

To test whether the identified upregulated genes were part of a bacteriome-specific response, we analyzed the expression of the same genes in TCT- or PBS-challenged carcasses of symbiotic insects as well as in TCT- or PBS-challenged aposymbiotic L4 (i.e., insects artificially devoid of symbionts, with no bacteriome, see the “Methods” section). We found that all eight upregulated AMPs (Fig. 2) and the other three upregulated genes (Fig. 3) were also induced in TCT-challenged symbiotic carcasses and TCT-challenged aposymbiotic whole larvae. The steady-state gene levels in PBS injection were comparable between the three conditions, with the exception of *lux*, *dpt-3*, and *srx*. Finally, in agreement with previous studies, these data show that the bacteriome induction is generally milder than the systemic response [20] but confirms the involvement of the bacteriome in the host immune response. Previous studies have shown that *colA* is chronically expressed in the larval bacteriomes, here seen at ~ 250 transcripts per million (TPM) in control conditions, and it successfully prevents endosymbiont escape and morphology [18, 19]. The TCT-induced AMP upregulation in the bacteriomes might therefore constitute a threat for endosymbiont fitness. Overall, these results strongly suggest that the presence of *S. pierantonius* does not affect the systemic induction of AMPs, which is comparable between symbiotic and aposymbiotic insects.

It is important to note that the present study failed to detect a couple of host genes previously identified as upregulated upon bacterial infection in *S. oryzae*, including the regulatory gene *pirk* and the Toll pathway-related genes (*pgrp*, *toll*), among others [20]. These discrepancies might indicate the inability of the TCT molecule to trigger a complete immune response, as opposed to a whole bacterium. TCT is a monomeric form of DAP-type PG and induces only the IMD and not the Toll pathway [19]. Nevertheless, the AMP induction observed here is consistent with previous studies [20, 24] and would be expected to constitute a severe threat for the endosymbionts in the absence of protective mechanisms.

### Symbiotic bacteria are insensitive to the activation of the bacteriome immune system

In order to identify potential signatures of bacterial stress and gene modulations to counteract the insect immune response and AMP induction, the symbiont transcriptomic profile obtained by dual RNA-seq from TCT-challenged bacteriome samples was compared with controls, i.e., PBS-injected or naïve. Remarkably, the differential analysis revealed that bacterial transcription is unresponsive to the TCT challenge (Additional file 3: Tables S5 and S6). Furthermore, and similarly to coding regions, we did not detect changes in the expression in repetitive regions (IS) (Additional file 3: Table S7). Moreover, a previous study using dual RNA-seq in *S. oryzae* showed around 400 differentially expressed bacterial genes throughout the metamorphosis of the insect, confirming the ability of the endosymbiont to modulate gene expression in response to host developmental stimuli [16]. The contrast between large changes of gene expression during metamorphosis, with a complete lack of differentially expressed genes upon TCT challenge, strongly suggests that the bacteria do not sense the AMP induction or any other stress induced by such challenge [40].

Rather, analysis of the complete bacterial transcriptome from both controls and TCT-challenged larvae displays similar gene expression. Highly expressed bacterial protein-coding genes detected within the bacteriome (Additional file 3: Table S8) are mainly involved in transcriptional regulation, translation, stress response, and virulence (see Additional file 2: Text S1 for more information [3, 15, 16, 18, 41–49]). Several transcriptional, translational, and stabilization factors of the general stress response sigma factor RpoS (reviewed in [50]) were similarly expressed at varied levels in all conditions (Additional file 3: Table S9). The expression of *rpoS* was lower than the vegetative sigma factor *rpoD*, which is a typical profile of the exponential growth phase in *Escherichia coli* [51]. This basal level of *rpoS* is also needed for triggering a fast stress response in diverse bacteria [50] and shows the ability of *S. pierantonius* from larval bacteriomes to quickly enter a “virulent mode” in the subsequent pupal stage that allows them to exit bacteriocytes and re-infect stem cells [16].

Together with previous findings that the symbiont population remains unchanged even after an immune challenge with pathogenic bacteria [20], this suggests that other regulatory mechanisms are in place to maintain the physical integrity of the symbiotic bacterial population during host AMP induction.

### Mature AMPs are physically separated from endosymbionts

**Fig. 4:**
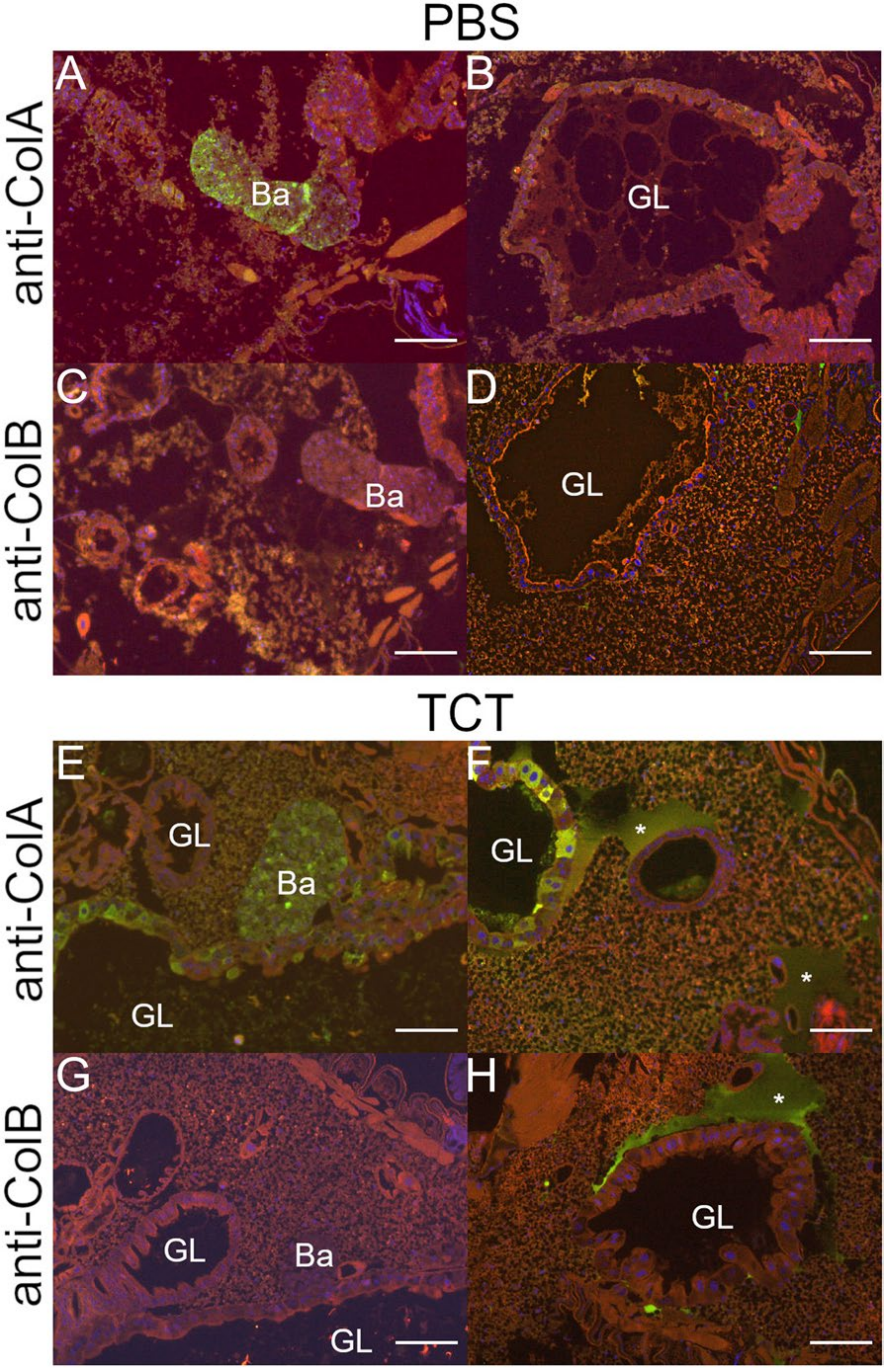
AMP localization in *S. oryzae* larvae, before and after TCT immune challenge. ColA (**A-B**) and ColB (**C-D**) localization in PBS-injected larvae. ColA (**E-F**) and ColB (**G-H**) localization in TCT-injected larvae. Ba, bacteriome; GL, gut lumen. Asterisks indicate accumulation of AMPs in the hemolymph. Scale bar, 50 μm

One of the hallmarks of AMPs is the presence of a N-terminal secretory sequence that addresses them to the outside of the cell, including the hemolymph, to counteract systemic infections [52]. Thus, even though cells in the bacteriome can produce AMPs, their final localization outside of bacteriocytes would ensure the protection of the endosymbionts from AMP harm. However, in physiological conditions, ColA is produced by and retained inside the bacteriocytes, together with the endosymbionts, where it keeps them from escaping [18]. Since our knowledge of AMP localization is still limited because of the lack of specific antibodies, it cannot be excluded that other AMPs might also accumulate intracellularly, especially if highly expressed, and constitute a threat to the endosymbionts. We therefore assessed the localization of TCT-induced AMPs with respect to the symbionts. We performed immunohistochemistry with polyclonal antibodies able to recognize *colB*, an AMP previously shown to be induced by TCT and bacterial challenges [19] and the bacteriome-specific AMP ColA [18]. The choice of *colB*, in particular, was dictated by the fact that, despite this peptide being very similar to *colA* (46.72% of amino acid sequence identity), their function is remarkably different, as *colA* is expressed constitutively in the bacteriome where it interacts with GroEL and contributes to the insect-bacteria homeostasis. Samples were taken at 6 h after the immune challenge with TCT or PBS (as for the transcriptomic analysis), so that we could confirm that AMPs were induced at the protein level, despite the lack of endosymbiont response. In the PBS-injected controls (Fig. 4A–D), ColA was detected within the bacteriome (Fig. 4A)—as expected because of its role in preventing symbiont escape [18] —but not in the other tissues (Fig. 4B). These results confirm the presence of ColA within the bacteriome, even at basal expression levels. In contrast, ColB was not detected inside the bacteriome (Fig. 4C), nor in other surrounding cells, including gut tissues (Fig. 4D). In response to TCT (Fig. 4E–H), ColA was still clearly detectable within the bacteriome (Fig. 4E), as expected, but also in several epithelial gut cells as well as in the acellular extended region that likely corresponds to the hemolymph (Fig. 4F). This confirms the dual role of ColA in both symbiosis control [18] and in response to an exogenous immune challenge. On the contrary, ColB was still absent from the bacteriome tissue following TCT challenge (Fig. 4G) but, similarly to ColA, was detected in the hemolymph of TCT-challenged larvae (Fig. 4H).

The results show that, in agreement with the lack of endosymbiont transcriptomic response, the excess of bacteriome-produced ColA, ColB and potentially all other AMPs (whether induced in the bacteriome or the fat body), remain physically separated from the endosymbionts. Thus endosymbiont integrity is protected even while AMPs participate in the systemic immune response.

## Conclusions

There are currently three main known strategies allowing symbiotic microorganisms to coexist with efficient and responsive insect immunity: (i) evolution of the ability to differentiate between pathogenic and symbiotic MAMPs by the host; (ii) bacterial molecular modifications leading to immune tolerance, notably promoting biofilm formation [53]; and (iii) compartmentalization of the symbionts in specialized symbiotic organs, often called bacteriomes [54]. The compartmentalization strategy sequesters the symbionts in specialized cells, creating a favorable environment for their metabolic activity and keeping them under control while avoiding overproliferation and virulence. The bacteriomes are therefore found in many insect species, including aphids [55], planthoppers [56], cicadas [57], and beetles [58]. Although very common, little is known about the evolution and immune modulation inside the bacteriomes, as well as their formation and maintenance.

**Fig. 5:**
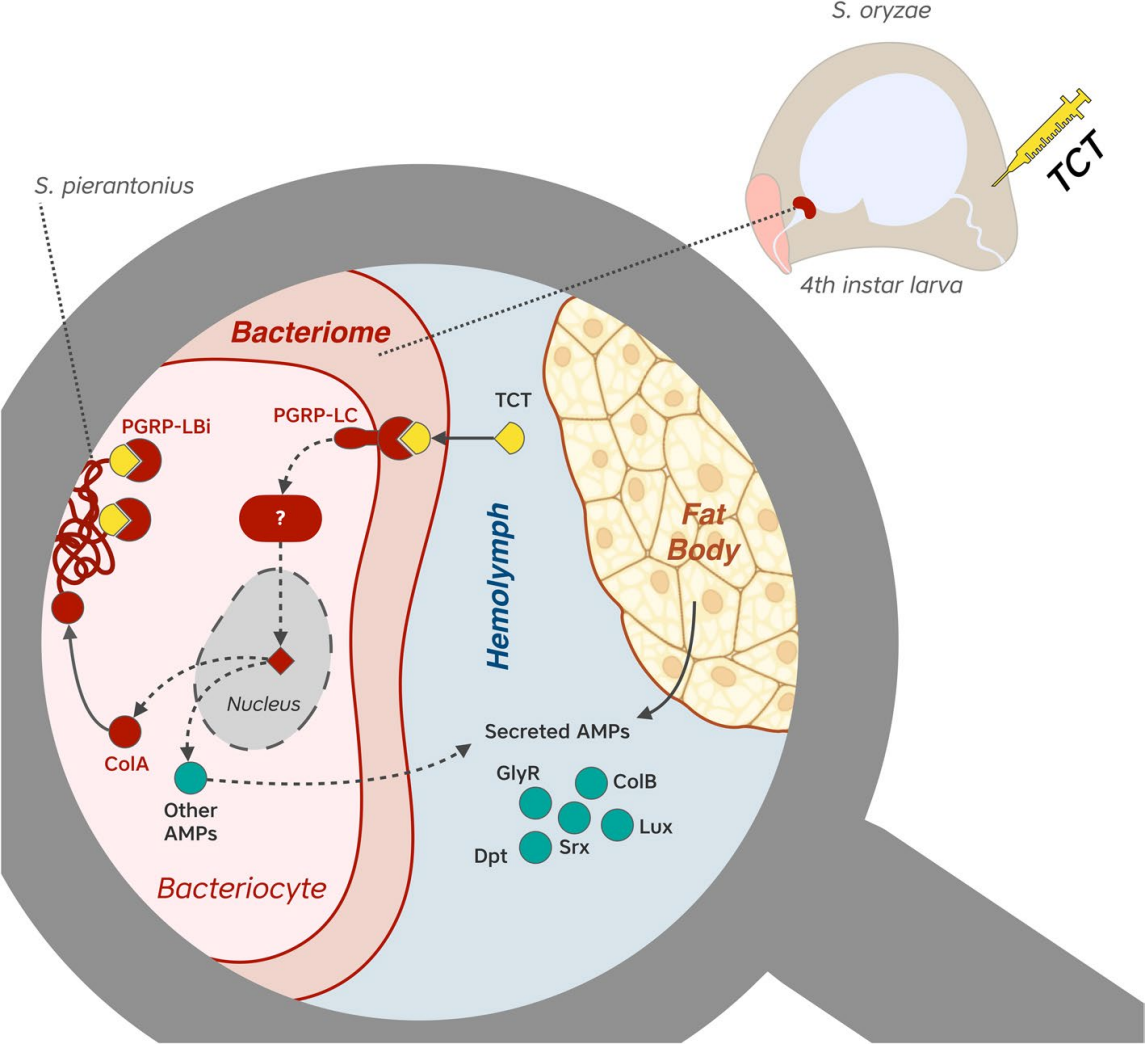
Proposed mechanisms of TCT challenge response within bacteriomes of *S. oryzae*. TCT injected in the hemolymph reaches bacteriomes and is recognized by PGRP-LC from bacteriocytes. Through a signaling cascade potentially dependent on IMD/RELISH proteins, bacteriocytes activate an AMP induction which for the most part are thought to be secreted (ColB, Srx, Lux, Gly-rich AMP, Dpt-2, Dpt-3, and Dpt-4) to aid in the global immunity response, but no effectors are perceived by the bacteria within bacteriomes. AMPs are also produced by the fat body and secreted to the hemolymph. ColA in turn is kept within bacteriocytes to prevent *S. pierantonius* from exiting the host cells during this immune challenge

In the *S. oryzae*/*S. pierantonius* symbiosis, bacterial MAMPs are able to trigger a potent immune response, thus excluding a selective tolerance of the weevil immune system towards *S. pierantonius* MAMPs [17, 19–21]. The absence of bacterial transcriptomic response to the systemic TCT immune challenge excludes active mechanisms of immune suppression from the endosymbiont. Rather, compartmentalization of *S. pierantonius* within bacteriomes guarantees physical separation between the endosymbionts and AMPs that might be produced by the bacteriome itself or elsewhere (e.g., fat bodies). This mechanism is crucial to protect both the host from the symbionts and the bacteria from the insect immune system [18, 21]. As demonstrated by the immunofluorescence labeling, there is no colocalization of endosymbiont-containing cells and AMPs, with the notable exception of ColA due to its homeostatic function, thus showing that not only the bacteriome acts as a physical barrier against the external AMPs, but is also capable to efficiently drain away the toxic molecules produced both inside or outside the bacteriome (Fig. 5). Altogether, these data refine the understanding on how an organ such as the bacteriome can ensure specific symbiotic function, i.e., maintain and control endosymbionts in a specific location, while potentially participating in the immune response to exogenous bacteria.

## Supplementary Information

**Additional file 1: Fig. S1.** Differential expression of TCT-repressed genes in bacteriomes, according to Dual RNA-seq. The quantification was performed by qRT-PCR on *S. oryzae* bacteriomes and carcasses of symbiotic weevils, as well as on whole aposymbiotic larvae. Green dots: PBS-injected larvae (control); red squares: TCT-injected larvae.

**Additional file 2: Text S1.** Description of genes highly expressed *from S. pierantonius*.

**Additional file 3: Table S1.** Primer sequences. **Table S2.** Dual RNA-seq trimming and mapping statistics. **Table S3.** Count data of *S. oryzae* genes. **Table S4.** Differential expression analysis of *S. oryzae* genes. **Table S5.** Level of expression of *S. pierantonius* genes. **Table S6.** Differential expression of *S. pierantonius* genes. **Table S7.** Count data and differential expression analysis of *S. pierantonius* ISs. **Table S8.** Highly expressed bacterial genes in all conditions (TPM > 1000) belonging to key biological functions in *S. pierantonius*. **Table S9.** Expression levels of genes related to the general stress response in *S. pierantonius*.

## Data Availability

Sequencing data from this study have been deposited at the National Center for Biotechnology Information Sequence Read Archive, https://www.ncbi.nlm.nih.gov/sra (accession no. PRJNA816415).

## Abbreviations

AMP: Antimicrobial peptide
BSA: Bovine serum albumin
cDNA: Complementary DNA
DAPI: 4,6-Diamidino-2-phenylindole
EtOH: Ethanol
IMD: Immune deficiency
IS: Insertion sequence
L4: Fourth instar larvae
MAMPs: Microbial-associated molecular patterns
p-adj: Adjusted *p*-values
PBS: Phosphate-buffered saline
PFA: Paraformaldehyde
PG: Proteoglycan
T3SS: Type III secretion system
TA: Toxin-antitoxin
TCT: Tracheal cytotoxin
TPM: Transcripts per million
