# Supplementary figures and images for: Efficient compartmentalization in insect bacteriomes protects symbiotic bacteria from host immune system
Source: Microbiome. 2022 Sep 27;10:156. doi: 10.1186/s40168-022-01334-8 (PMC9513942; doi:10.1186/s40168-022-01334-8)

N/A (LOC115882681)

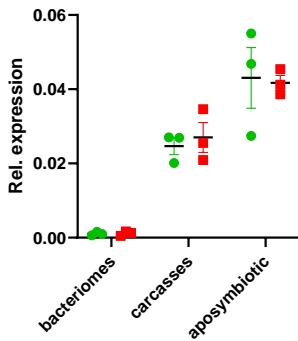*adf-1*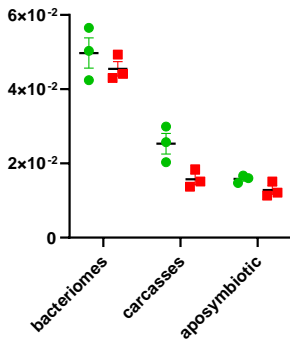*eif-4/ebp-2*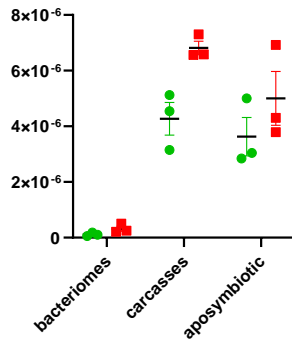*nrbp*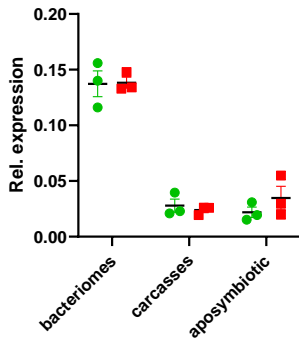*znf-91 like*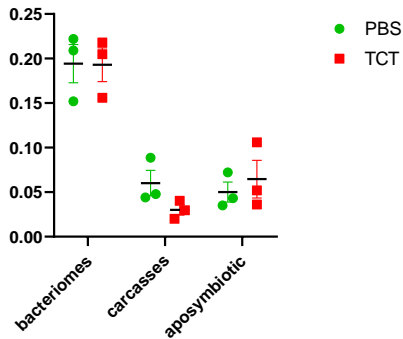

● PBS  
■ TCT

Supplement: Supplementary file 2 — Additional file 1: Fig. S1. Differential expression of TCT-repressed genes in bacteriomes, according to Dual RNA-seq. The quantification was performed by qRT-PCR on S. oryzae bacteriomes and carcasses of symbiotic weevils, as well as on whole aposymbiotic larvae. Green dots: PBS-injected larvae (control); red squares: TCT-injected larvae. [file 40168_2022_1334_MOESM1_ESM.pdf]
